# An International Comparison of Attitudes Toward Traditional and Modern Medicine in a Chinese and an American Clinic Setting

**DOI:** 10.1093/ecam/nen065

**Published:** 2011-06-23

**Authors:** Adam Burke, Tony Kuo, Rick Harvey, Jun Wang

**Affiliations:** ^1^Institute for Holistic Health Studies, San Francisco State University, San Francisco, CA 94132, USA; ^2^Department of Family Medicine, David Geffen School of Medicine at UCLA, 50-078 Center for Health Sciences, Los Angeles, CA, USA; ^3^Department of Public Health, Los Angeles County, Los Angeles, CA, USA

## Abstract

*Introduction*. International comparative research on traditional medicine (TM) offers a useful method for examining differences in patient characteristics and can provide insight into: (i) more universal characteristics which may cross cultures and international borders; (ii) unique characteristics influenced by regional/national factors; and (iii) cultural values of immigrant populations. To explore these issues TM patients from the United States and China were compared. *Methods.* Data collection took place at two TM college clinics. A convenience sample of 128 patients in China and 127 patients in the United States completed a 28-item questionnaire. *Results.* There was a marked similarity between the two patient groups in terms of the biological characteristics of age and gender. Musculoskeletal issues were the most common presenting complaints in the United States; while in China TM was used for a more diverse array of conditions. The majority of patients in both countries had initially used allopathic medicine (AM); significantly, more of the United States respondents stopped allopathic treatment after beginning traditional treatment. In comparing the two countries, patients in China were significantly more satisfied with AM and American patients significantly more satisfied with TM. In comparing the two medicines, the patient samples in both countries were significantly more satisfied with TM than AM. *Discussion.* Although treatment often originated with allopathic providers, many patients sought alternatives presumably to find the best solution to their problems. This tendency toward self-assignment suggests that a pluralistic healthcare system may provide the greatest satisfaction resulting from personal choice and improved outcomes.

## 1. Introduction

National surveys have shown growing use of complementary and alternative medicine (CAM) by the general public [1–4]. Others have reported on the nature of use by specific populations, such as by disease [HIV/AIDS [5, 6] pain [7, 8] and cancer [9–11]], race/ethnicity [12, 13] or gender [14, 15]. More recently, increased attention has been focused on specific CAM therapies, including traditional East Asian medicine (TM) [16–19]. These studies have provided important information on demographics and the nature of use. Less common are studies examining why individuals elect to use specific CAM therapies, including studies that evaluate attitudes, beliefs and other sociocultural and psychosocial correlates of use, especially in relation to allopathic medical care. Such information would shed light on why consumers seek alternatives outside of conventional care, whether such alternatives independently or in conjunction with conventional care produce improved outcomes (on a variety of levels), and what characteristics identify individuals who choose alternative methods from those who do not. International comparative CAM/TM research offers a useful method for examining differences in these patient characteristics. International comparisons have the capacity to provide insight into: (i) more universal characteristics which may cross cultures and international borders; (ii) unique characteristics influenced by regional/national factors such as local custom, culture specific health beliefs or determinative aspects of a geographic region (e.g., climatic or economic development differences); and (iii) cultural values of the immigrant population, which may be useful for improved understanding of healthcare service needs and barriers. To date no such direct international comparative CAM/TM studies have been conducted to examine such issues.

One CAM modality that merits international comparison is TM. TM is comprised of acupuncture, herbal therapy, qigong, physiotherapy (tui na), moxibustion and other methods. It is a provider-based CAM modality able to treat a wide variety of health concerns, consequently offering a viable alternative to allopathic medicine (AM) in many instances. TM has an important historic and cultural legacy, with use in China since at least the Shang Dynastic period (17th to 11th century BCE). Acupuncture and related practices are among the more common “alternative medicine" methods in use globally [17, 20–22]. Acupuncture is also becoming an increasingly important aspect of multidisciplinary and integrative medical practice for use in pain management [23, 24], oncology [25], addiction treatment and other health problems in the United States [26–28]. Approximately 4% of the adult US population has tried acupuncture [1, 17]. Studies on TM/CAM conducted in the United States have also shown significant use by Asian women and by Chinese immigrants, with reported utilization rates among Southeast and East Asians ranging as high as 66–93% and annual use of acupuncture by Asian females being approximately three times higher than the national average [16, 17, 19].

Although a large body of research has emerged looking at the clinical safety and efficacy of acupuncture and herbs, information on attitudes and motives for use of CAM/TM is less developed, especially in relation to use of AM. One national random telephone survey of 831 respondents who saw a medical doctor suggested a pragmatic motive for CAM use. The study reported that the majority of respondents believed the combination of CAM and conventional treatments was superior to either alone, and that CAM use did not reflect dissatisfaction with conventional care [29]. Another national sample survey of 1035 respondents also evaluated motives for CAM use [30]. The author reported that the use of alternative therapies reflected a holistic orientation to health and that it was not significantly associated with dissatisfaction. Yet, numerous studies have reported on the observed association between patient dissatisfaction with AM and the use of alternative therapies [31–36]. The largest survey of CAM use in the United States conducted by the Centers for Disease Control and Prevention (CDC) found that 28% of respondents used CAM because they did not believe conventional medicine would help their condition. The authors concluded this finding suggested that patient dissatisfaction was an important contributor to CAM use [1]. Issues related to patient dissatisfaction reported in the literature include a consumer desire for more patient-centered care, more time with providers, more holistic interventions, less institutional and technological health services, more personal control and active participation in treatment and health maintenance, therapies that produced fewer side-effects, more effective treatment and greater choice [33, 36–42].

It is possible that the observed association between dissatisfaction and CAM use may be more typical in countries such as the United States, where CAM methods such as acupuncture enjoy the benefit of novelty relative to AM. A comparative study of attitudes toward TM and AM with patients in the United States and an East Asian country could help clarify this issue. To date, no TM studies have directly compared differences in patient demographics, patterns of utilization, beliefs or attitudes between Mainland China and the United States. To provide additional information on dissatisfaction and related issues an international comparative study was conducted. It was hypothesized that patients from the United States and Mainland China would be similar in terms of biological characteristics (e.g., age and gender) and presenting complaints, but that they would differ in their attitudes toward TM and AM.

## 2. Subjects and Methods

### 2.1. Study Sites

The study was conducted at the Chengdu University of Traditional Chinese Medicine (CDUTCM) and the American College of Traditional Chinese Medicine (ACTCM). CDUTCM is located in Chengdu, Sichuan Province, China. Chengdu is a city of over 10 million population, and the capital of Sichuan. CDUTCM is one of the five founding colleges of TM in China. Given its distance from the more highly westernized cities on the eastern seaboard, such as Shanghai and Beijing, CDUTCM has maintained a strong commitment to traditional medical practices versus a more integrative model adopted in some other TM colleges.

ACTCM is located in The San Francisco Bay Area, a region of 7 million people, is a major center for alternative medical practice and thought, and has one of the largest populations of ethnic Chinese in the United States. ACTCM was founded in 1980 and is one of the pioneer training programs in the United States. A review of demographic data from a sample of 570 recent ACTCM patients found them to be 61% female, an average age of 40.2 years, 62% White and 15% Asian, with a predominant educational attainment of college 59% or graduate school 32%. Equivalent data were not available from CDUTCM, but estimates of typical patients would be individuals in their mid-40s, and a higher percentage female.

Both CDUTCM and ACTCM train TM providers and offer a full range of clinical services to patients. CDUTCM offers both outpatient services and in-service care in its 600-bed teaching hospital. ACTCM provides outpatient services exclusively. The institutions are similar in the types of outpatient treatments offered and they both rely on shared TM views of etiology, diagnosis and treatment. The ACTCM faculty includes 18 traditional doctors from China, with 3 of those from Chengdu. The two colleges also have a “sister" school relationship providing an opportunity for students from ACTCM to do a rotation in a large traditional hospital in China.

### 2.2. Participant Recruitment

Participants were selected from a convenience sample of patients who appeared for treatment on the days of data collection during the summer of 2002. Individuals had to be at least 18 years of age and able to complete the survey. Recruitment was done in the waiting areas. CDUTCM data were collected in the herbal pharmacy waiting area, and in ACTCM's reception/pharmacy waiting area.

### 2.3. Data Collection Procedures

Data were collected at CDUTCM by two interviewers (the primary author and a translator; the translator was a CDUTCM-trained traditional doctor with excellent medical English language skills). Prospective participants were informed of the purpose of the survey and consent was obtained. Approximately 12% of those solicited did not participate. At ACTCM, data collection was conducted by the primary author and trained office assistants. About 10% did not participate. Common reasons for non-participation included expecting to be called for appointments, and feeling ill.

### 2.4. Survey Instrument

A 28-item questionnaire was used to collect information on demographics, health status, nature and duration of presenting health complaints, use of and attitudes toward TM and AM and perceived efficacy of and satisfaction with both medicines. The questionnaire was based on a longer 88-item instrument developed by the principal author and tested on approximately 200 patients at ACTCM in a previous related study. Key themes and items were derived from this earlier survey. The 28-item questionnaire was pre-tested in English with acupuncturists and patients and translated into Chinese by a professional translator. It contained 25 closed-ended and 3 open-ended questions. In China, translation of open-ended items back into English was done each day by the first author and the CDUTCM translator.

The San Francisco State University Office of Human Subjects Protection approved the study. Incomplete surveys, 5 in the US group and 13 in the China group, were discarded. This left complete data from 127 participants in the United States, and 128 in China. Descriptive statistics and non-parametric comparisons of the two groups were performed on key measures. Tests for significant differences between groups were conducted using Mann-Whitney U-test and the Wilcoxon signed-ranks test statistics.

## 3. Results

### 3.1. Participant Sociodemographics

San Francisco participants (*n* = 127) were on average 42.4 years of age (range 23–80) (Table 1). They were more likely to be female, White, single and with some college education. In Chengdu, the average participant (*n* = 128) was 44.4 years of age (range 18–86), and more commonly female, Chinese, married, with a high school education.

### 3.2. Health Concerns

**Figure 1:**
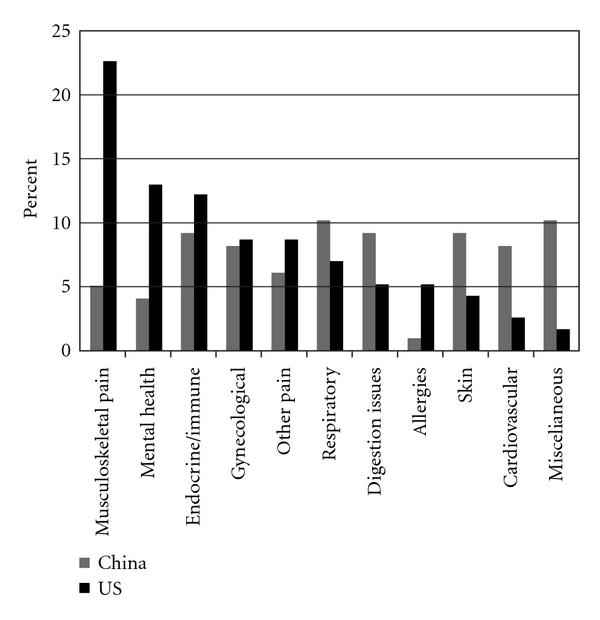
Most common presenting complaints for TM by country.

The US participants were more likely to describe their general level of health as “good" as compared to “fair" for the China sample. Chronic health problems were the major reason for TM use in both countries, but more individuals in China used TM for acute symptoms compared to the United States. Significantly, more of the US sample reported initially seeking allopathic medical treatment for their health problems compared to the China sample (81% versus 69%; *P* = .031). Regarding continued use of AM, significantly more of the China sample continued AM once they started receiving TM treatment for their complaint (60.3% versus 41.6%; *P* = .006). When asked about the reason for seeking care, musculoskeletal issues were the most common presenting complaint in the United States (Figure 1). In China, the most common presenting complaints included respiratory, miscellaneous, skin, digestive, immune/endocrine and pain.

### 3.3. Attitudes toward AM and TM

A comparison of US and China patient's attitudes toward AM and TM is provided in Table 2. Participants in both countries were asked to rate the medical treatments they received in terms of perceived efficacy and satisfaction. A 5-item Likert-type scale was provided with the following choice options: 0—very low, 1—low, 2—moderate, 3—high or 4—very high. US respondents perceived AM efficacy as “low" compared to “moderate" in China (*P* < .001). Similarly, satisfaction with AM in the United States was significantly lower than for the China sample (*P* < .001). When asked if dissatisfaction with AM had been a motivating factor in the decision to use TM, there was an equivalent “high" agreement in both the United States and China (*P* = .173). Participants also rated their experience with TM in terms of perceived efficacy and satisfaction. For both the US and China samples, the level of perceived efficacy was “high" on average. Although high in both countries, reported satisfaction was significantly higher in the United States than in China (*P* < .001). The US participants also reported that their intention to continue using TM was “very high" compared to “high" in the China sample (*P* < .001).

When comparisons were performed between the two medicines, participants in the United States and China perceived TM as being more effective at treating their presenting complaint than AM (United States,*P* = .001 and China, *P* = .03) (Table 3). Similarly, in both countries patients were more satisfied with TM than with AM (United States, *P* = .001 and China, *P* = .001).

### 3.4. Positive and Negative Attributes of TM

Participants responded to several open-ended questions to state what they liked and disliked about TM. For the US sample, the most common positive attributes mentioned included that it was: holistic, balanced and natural; delivered in a caring way; effective; and produced few side effects. For the China sample, the most commonly reported positive attributes included that is was: holistic, natural and balanced; cured the root; was effective; and produced few side effects. The most common complaints in the United States were discomfort from the needles, the taste of herbs and the lack of insurance coverage. In China, the most common complaints were slow effect, the inconvenience of cooking herbs and the taste of herbs.

## 4. Discussion

### 4.1. Gender and Race

Consistent with the original hypothesis, the samples of participants from the two countries had highly comparable demographic characteristics specifically in terms of age and gender, what Hulka and Wheat termed the “biological imperative" for healthcare [43]. Indeed, in both countries use was more common for females and mid-aged patients. Age and gender have been shown to be related to CAM and TM use in the United States (mid-aged and female) [3, 4, 15, 17, 44]. This may reflect a transcultural aspect of acupuncture/TM/CAM usage. For race, the San Francisco sample was predominantly White followed by Asian. Asians and Whites have been found to be the largest proportional user groups of CAM/TM by race/ethnicity in representative US surveys (when prayer for health and megavitamin use are excluded from the definition of CAM) [1, 17]. The Chengdu sample was 100% Asian (Chinese).

### 4.2. Education

The China sample had a lower level of educational attainment compared to the United States. This could reflect the existing differences in education in the two countries despite China's dramatic improvements in literacy and compulsory education of the last 50 years [45]. It may also reflect the relationship between use and education. Numerous US studies have found a positive relationship between education and CAM/TM use [1, 13, 17, 30, 46, 47]. However, other studies focusing more specifically on Southeast and East Asian American populations have found that individuals with lower levels of educational attainment, lower SES or limited English proficiency, were very likely to use their own culturally relevant TMs (as distinct from more general CAM usage) [16, 17, 19]. Relatedly, studies in China examining attitudes toward TM reported a more favorable attitude toward TM by individuals who were older, less educated or those holding traditional health beliefs [48–50]. These findings may suggest cultural differences in the use of TM related to educational attainment, with the adoption of the “new" TM being associated with more education in the United States while the maintenance of “old" TM in China associated with less education.

### 4.3. Health Concerns—Musculoskeletal Pain

The primary reason reported for use of TM in both countries was to address chronic health problems. This has been observed in other CAM studies [7, 14, 30, 51]. However, contrary to the original hypothesis the presenting complaints differed in the two countries. In the United States, the top complaints treated were musculoskeletal, emotional/mental health, immune/endocrine, other pain and gynecological issues. Musculoskeletal pain was notably higher than the others (22.6% of respondents). This finding matched the results of other studies in the United States showing CAM/TM use to be significantly related to the treatment of chronic pain, especially back pain [30, 52, 53]. In the China sample, no single complaint stood out as predominantly as musculoskeletal pain in the United States. Other studies in Asia have reported high use for musculoskeletal complaints, such as a review of national acupuncture outpatient insurance claims reported by Chen in Taiwan [54]. Napadow and Kaptchuk in an observation of two major acupuncture outpatient clinics in Beijing did not find musculoskeletal complaints to predominate, but rather neurological conditions, and suggested that local disease prevalence and patient expectations were major contributors to differences in regional use [55]. It must be noted, however, that both of these studies focused on acupuncture compared to TM. The later is a more comprehensive form of treatment that includes herbs and other modalities in addition to acupuncture. In contrast to these studies, the CDUTCM data were collected in the herbal pharmacy waiting area of the hospital, rather than in outpatient acupuncture. Individuals waiting for herbs may or may not have received acupuncture as part of their treatment. Care at ACTCM was similarly comprehensive for most patients, including acupuncture, herbs and additional modalities as needed (which reflects the training, treatment style and non-hospital-based practices of the majority of US providers offering East Asian TM services) [56]. If data had been collected in the CDUTCM outpatient acupuncture area, more musculoskeletal or neurological issues would have been observed as noted in these other studies. Neurological and musculoskeletal complaints, such as Bell's Palsy, MS and musculoskeletal pain, were common issues treated in CDUTCM outpatient acupuncture.

### 4.4. Other Health Concerns in China

Of the presenting complaints reported in Chengdu, several stood out. First, respiratory symptoms were common (10.2%). This may reflect local disease prevalence; Chengdu is ranked as one of China's top cities for air pollution [57]. This would support Napadow and Kaptchuk's [55] notion of local disease prevalence influencing treatment patterns. “Miscellaneous" was another interesting symptom category (10.2%). A large national study of acupuncture use in Taiwan similarly found “ill-defined conditions" to be one of the major treatment categories [54]. In Chengdu, this group of patients sought care for diverse symptoms including dizziness, itchy eyes and sensitivity to salty food. Following those two categories, there were comparable levels of reported usage for symptoms related to skin conditions, digestive system complaints, endocrine/immune disorders, cardiovascular disease, gynecological issues and pain (including musculoskeletal and other pain, such as migraine headaches). Using TM for such a diverse array of conditions may reflect the knowledge and cultural beliefs of patients in China, seeing TM as a primary care modality, used as such for millennia, and therefore viewed as a viable option for many medical conditions, especially those of a chronic nature. Also, although less common a number of patients also sought treatment of symptoms based on patient-reported traditional medical etiologies, such as “damp heat" as has been reported in related anthropological studies [49, 58]. Such traditional medical descriptors are recognized by the general population and are standard TM diagnostic criteria.

### 4.5. Attitudes toward TM and AM

Significantly, more patients in the United States (81%) had initially visited allopathic medical providers before seeking TM care, compared with China (69%). Another survey conducted in the United States reported that of 411 respondents who reported seeing both CAM and conventional providers 70% had also seen an allopathic provider (before or concurrent with seeing a CAM provider) [29]. Although fewer Chinese initiated treatment with an allopathic provider, more of them continued using AM in addition to TM for treatment compared to the US sample. The Chinese sample also found AM to be more effective, and was more satisfied with AM compared to the US sample. These results may reflect the Chinese population's greater historic exposure to the two types of healthcare. As a consequence, Chinese patients may have been more confident in their healthcare decision-making regarding when to see an allopathic doctor or a traditional doctor, especially with both medicines being viewed as comprehensive systems of care. A number of the China patients in this study, for example, reported that TM “cured the root" rather than “treated the surface". This health belief, that TM is a more appropriate treatment for more persistent chronic conditions, has been observed in other ethnographic research [59]. If Chinese patients initially chose an allopathic provider for treatment, given this confidence in self-assignment, they could be expected to continue allopathic care and be more satisfied with it. Kleinman and others [60, 61] have suggested that patients in China tend to retain significant decision-making authority regarding their disease management.

### 4.6. Novelty and Positive Attribution of TM

Although TM has been present in the United States since the 1850s when large numbers of Chinese came to participate in the economic boom of the Gold Rush era, it has only become a visible force in healthcare during the last several decades [62]. By the second half of the 20th century, American culture was undergoing a significant cultural transformation which created a supportive milieu for alternative health practices [63]. In 1971, the Nixon administration was engaged in a historic diplomatic mission to strengthen relations with China exposing many Americans to Chinese culture, including acupuncture. Acupuncture was ultimately legalized in the United States in the 1970s and centers were opened to train Americans in its practice. During the last several decades, this “new" medicine has become an increasingly important element of care across a wide range of diseases and an important component of integrative medical services [23–28].

Although the Jesuits first introduced western medicine into China in the 17th century, it did not begin to have a notable impact on the culture until the mid-1800s. Its influence was heightened by the demise of dynastic rule in 1911. TM, seen as a vestige of the old order, was threatened with extinction during the ensuing period of social reform and modernization. Despite these pressures, it survived and experienced a significant revival after the creation of the People's Republic of China in 1949 [64–66]. More recently, AM's role has advanced significantly as the result in part from economic and social reforms instituted by Deng Xiao Ping in the 1990s. Many of these reforms have advantaged AM including the elimination of free universal healthcare, government hospital reimbursement plans favoring AM treatments, and significantly greater competition from diverse allopathic medical services. Globalization and modernization have also positively affected perceptions of the “new" medicine as a result of changing cultural values and lifestyles [20, 48, 49, 67].

As suggested in the hypotheses, dissatisfaction with AM and favorable attitudes toward TM in the West may have been a function of TM's novelty. Conversely, the perceived efficacy of and satisfaction with allopathic medical care in China may have similarly been a function of positive attribution due to novelty. Consumer research has shown that many buyers seek novel products and ideas, making novelty seeking and innovativeness important aspects of consumer decision-making and purchasing [68, 69].

Indeed, satisfaction with TM was significantly higher in the United States (where it was “new"), compared to China. Conversely, satisfaction with AM was significantly higher in China (where it was “new"). These results would suggest that the lower degree of satisfaction with AM in the United States and with TM in China could have resulted in part from a bias toward the “new" or more novel medicine. This reflects the old Chinese adage, that the foreign moon is always brighter. It remains important to note, however, that the higher rate of satisfaction with TM (compared to AM) in both countries still speaks to potentially unique benefits (cultural, physiological, psychological or other) which TM appears to provide to these individuals.

### 4.7. Limitations

There are a number of factors that limit the interpretation of findings including that the samples were small, non-random/representative, self-selected and that *∼*11% of those invited did not participate, reducing information on that sub-sample. Also, data were collected at comprehensive TM clinics. Patients receiving treatment at these sites may have been culturally or otherwise predisposed to TM and thus be more favorably inclined. One might expect different results if patients seeking traditional East Asian medical treatments were surveyed in large allopathic medical centers providing such services. Although such comprehensive programs do not exist, one could at least do a comparison with centers offering integrative programs, which often include acupuncture. This would be useful. Given the relative dearth and novelty of such centers, however, they too would presumably constitute biased samples. Despite the potential patient bias, it is useful to note that in both countries the majority started with allopathic care. In China, the respondents had more favorable attitudes toward allopathic treatments (compared to United States), but in both countries dissatisfaction with allopathic treatment outcomes were significant reasons for selecting TM care. Although methodological limitations are recognized, there were similarities between the study findings and those of large population-based CAM studies in the United States in terms of gender, age and disease chronicity, and also on attitudes and behaviors based on results from several extensive ethnographic studies on TM conducted in China.

## 5. Conclusions

Respondents in both countries indicated a high degree of efficacy and satisfaction with TM, compared to AM. Although most patients in this study initiated treatment with AM providers, many ultimately stopped AM treatment and sought alternatives, presumably in an effort to find what they believed to be the most appropriate and effective care. A number of factors, such as culturally relevant health beliefs related to the nature of disease and treatment, may have contributed to this migration. This tendency toward self-assignment suggests that a pluralistic healthcare model may provide greater satisfaction for consumers seeking solutions to complex health problems. Continued exploration of sociocultural and psychosocial factors related to the choice to use TM is urgently needed. Given TM's position as a viable alternative treatment modality, such exploration could deepen our understanding of what patients are hoping to find in their pursuit of health and well-being. Opportunities for insight may be lost as TM faces potential contraction within a modern healthcare marketplace [70].

## Figures and Tables

**Table 1:** Distribution of select participant characteristics.

Characteristics	United States (ACTCM)		China (CDUTCM)	
	Frequency	Percent	Frequency	Percent
Age (years)				
18–29	22	17.5	25	19.0
30–39	32	25.4	29	23.0
40–49	33	26.2	32	25.4
50–59	28	22.1	16	12.7
60–69+	11	8.8	25	19.9
Gender				
Female	78	61.9	77	61.1
Male	48	38.1	49	38.9
Race/ethnicity				
Asian	12	9.5	126	100.0
White	95	75.4	0	0.0
Other	19	15.2	0	0.0
Education				
Primary/high school	7	5.6	79	62.7
College	68	53.9	45	35.7
Graduate school	51	40.5	2	1.6
Marital status				
Currently married	20	18.5	105	83.3
Single no partner	54	50.0	2	1.6
Living with partner	23	21.3	1	0.8
Divorced/widowed	11	10.3	18	14.3
Health status				
Poor	9	7.1	25	19.8
Fair	28	22.2	72	57.1
Good	56	44.5	22	17.5
Very good	25	19.8	7	5.6
Excellent	8	6.4	0	0.0
Nature of health concern				
Chronic	97	85.1	77	72.0
Acute	17	14.9	30	28.0
Initial AM	81	39.4	69	46.6
Continue AM	42	49.5	60	49.1

**Table 2:** Comparing attitudes toward allopathic and traditional treatments between countries.

	United States (ACTCM)		China (CDUTCM)		
	Mean	SD	Mean	SD	*P* ^a^
Perceived efficacy of AM	1.42	1.11	2.08	0.97	<.001
Perceived efficacy of TM	2.73	0.78	2.51	1.06	.178
Satisfaction with AM	1.35	1.05	1.84	0.75	<.001
Satisfaction with TM	3.33	0.63	2.59	0.77	<.001
Dissatisfaction AM—use TM	2.50	1.38	2.67	1.44	.173
Intention to use TM future	3.65	0.51	2.95	1.17	<.001

^a^Mann-Whitney U-test.

**Table 3:** Comparing attitudes toward allopathic and traditional treatments within countries.

	Allopathic		Traditional		
	Mean	SD	Mean	SD	P*
Perceived efficacy (United States)	1.42	1.11	2.73	0.78	.000
Perceived efficacy (China)	2.08	0.97	2.51	1.06	.030
Satisfaction (United States)	1.35	1.05	3.33	0.63	.000
Satisfaction (China)	1.84	0.75	2.59	0.77	.000

*Wilcoxin signed-rank test.
